# Tuning crystallinity and electronic structure of Co_3_O_4_ nanoparticles *via* hydrothermal time for improved photocatalytic dye degradation and statistical kinetic evaluation

**DOI:** 10.1039/d6na00411c

**Published:** 2026-09-07

**Authors:** Elbadawy A. Kamoun, Habiba A. Hossni, V. Ganesh, Mohammed A. Alkhalifah, M. Y. Nassar, Esam M. Bakir, Heba Y. Zahran, Ibrahim S. Yahia

**Affiliations:** a Department of Chemistry, College of Science, King Faisal University Al-Ahsa 31982 Saudi Arabia ekamoun@kfu.edu.sa badawykamoun@yahoo.com; b Nanotechnology Section, Egyptian Company for Carbon Materials (ECCM) El-Sheraton/El-Nozha Cairo 11757 Egypt; c Laboratory of Nano-Smart Materials for Science and Technology (LNSMST), Department of Physics, Faculty of Science, King Khalid University P.O. Box 9004 Abha Saudi Arabia dr_isyahia@yahoo.com

## Abstract

This study explores photodegradation using needle-like Co_3_O_4_ NPs with time-dependent morphology, fabricated using a hydrothermal method with different hydrothermal reaction times (12 h, 24 h, 48 h, and 72 h), as a photocatalyst for organic dye degradation under simulated solar light irradiation. XRD, FTIR spectroscopy, SEM, EDX, diffuse reflectance spectroscopy (DRS), and BET analyses were used to characterize the prepared Co_3_O_4_ NPs. A notable temperature-dependent transition in crystallinity and phase composition, coupled with a notable reduction in lattice strain and refinement of the grain size at higher temperatures, was observed. The results demonstrated a decrease in band gap from 3.53 eV to 3.46 eV as the hydrothermal reaction time increased progressively, implying higher electronic structural ordering and improved light-harvesting capabilities. Selectivity studies on degrading the EY and BF dyes at 5 ppm showed distinct responses for catalysts with different Co_3_O_4_ preparation times. Co_3_O_4_ NPs synthesized over 48 h with 6.5 m^2^ g^−1^ loading exhibited high selectivity for EY and BF, with 71.2% and 76.9% degradation, respectively. Kinetics were analyzed using pseudo-first-order models and Minitab software to evaluate the experimental data and model the reaction behavior, with a statistically significant *p*-value of ∼0.000. Recyclability tests demonstrated that Co_3_O_4_ was highly stable under simulated solar light dye degradation conditions. The enhanced photocatalytic activity of Co_3_O_4_ NPs is likely due to the optimized hydrothermal reaction time that resulted in Co_3_O_4_ NP structures with reduced charge recombination and increased catalytic activity. The findings demonstrate the importance of hydrothermal reaction time for designing 48 h Co_3_O_4_ nanostructures with optimized structural features and better photocatalytic activity.

## Abstract 2

Synthesis route of Co_3_O_4_ NPs *via* different hydrothermal reaction times.

## Graphical abstract

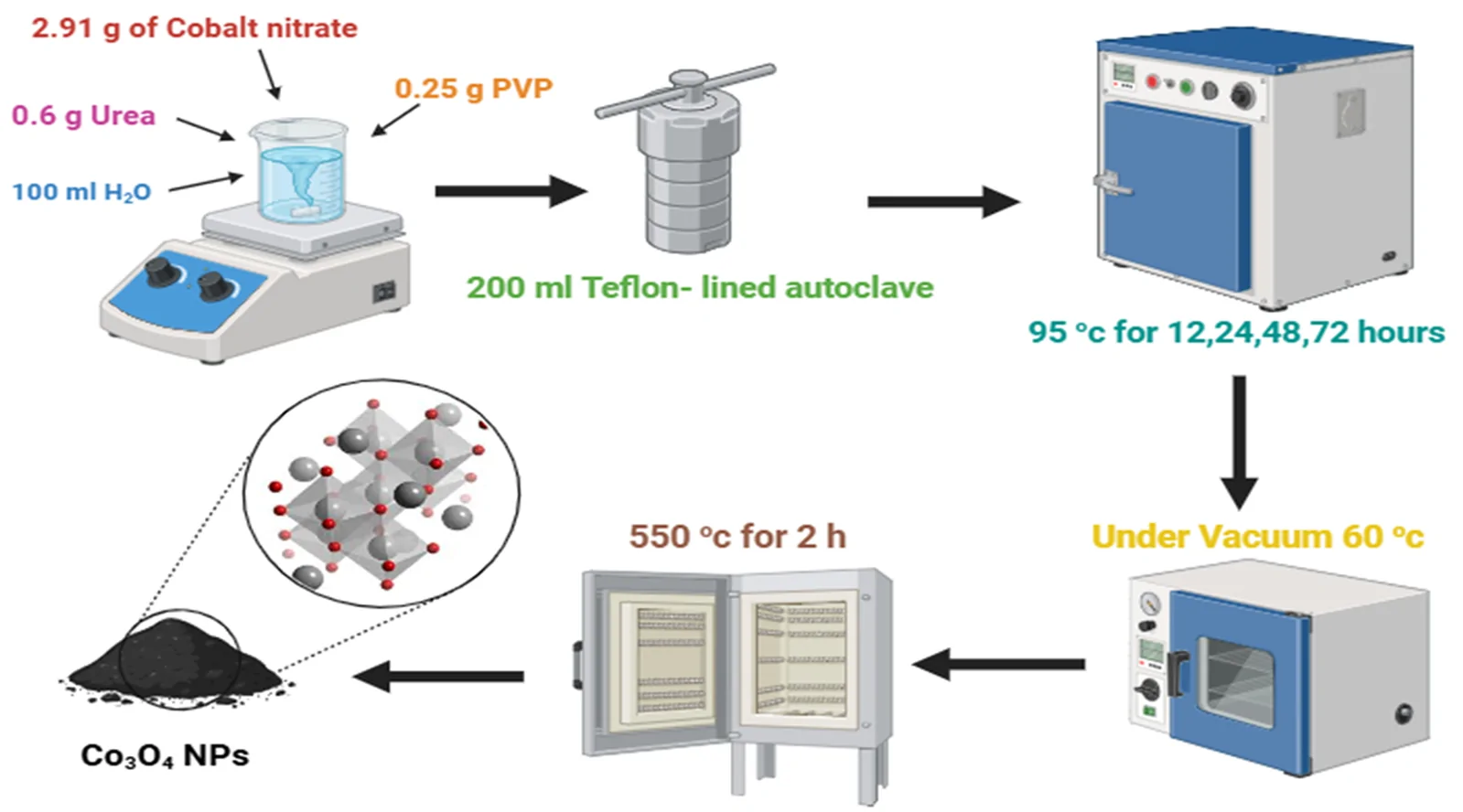

## 1 Introduction

Water pollution is a global problem that negatively impacts our health. Driven by increasing human activity, pollutants influence the complex chemical composition of natural water systems, posing risks to ecosystems and habitats as contaminant concentrations increase.^1,2^ Among various contaminants, the amount of wastewater generated from the use of dyes is on the rise, with more than 10^5^ different dyes produced annually, which generates large amounts of wastewater pollutants.^3^ Due to its complex composition, high concentration, rich color, and multiple biodegradable components, wastewater generated from dye production is challenging to treat.^4^ Especially, untreated dyes pose a threat to the environment and human health. As a result, before releasing industrial waste into the environment, it must be completely treated.

Pharmaceutical dyes are used in medication formulations and medical diagnostics; however, these dyes are toxic, unstable, and persistent in aquatic environments and pose significant environmental risks as they damage aquatic ecosystem photosynthesis, endanger human health, and degrade water quality. Moreover, some dyes can harm the liver and kidney tissues, pose a danger from long-term exposure and possibly be genotoxic.^5–7^ Due to the high cost and inefficiency of traditional wastewater treatment methods, sustainable alternatives such as advanced photocatalysts and adsorption are being investigated.^8^ These types of pollutants are better removed by non-traditional methods that convert them into harmless chemicals without producing hazardous byproducts.

Pollutants can be efficiently removed by advanced treatment technologies including physical, chemical, and biological techniques. One of the most challenging modern techniques is the advanced oxidation process, which uses nanocatalysis to convert hazardous pollutants into non-toxic inorganic molecules.^9,10^ When a photon of sufficient energy is absorbed, the catalyst accelerates the photoinduced process, known as photocatalysis. A hole is then created in the valence band as a result of charge separation. The goal is to prevent electron–hole recombination. The electrons produced by photocatalysis can either reduce the dye or react with electron acceptors to produce radicals; the radicals can also be generated when organic molecules are oxidized by photogenerated holes.^11^ However, the photocatalytic performance of newly developed photocatalysts needs to be improved to satisfy the demands of real-world applications. The use of semiconductor nanoparticles offers an environmentally friendly option for dye degradation. A variety of photocatalysts have been studied, such as metal oxides, dye-doped oxides, and nanocomposites.^12,13^ Metal oxides, *e.g.* TiO_2_, ZnO, CuO, FeO_3_, and Co_3_O_4_, are being extensively researched for dye degradation because of their availability, affordability, chemical stability, and suitable band gap. They produce pairs of electrons and holes, which decompose complex color molecules into innocuous byproducts.^14,15^ However, their efficiency is decreased by rapid recombination. Doping and the use of oxide-based heterojunctions are two techniques that enhance charge separation.^16^ The type of material and the manufacturing process have significant impacts on photoactivity. The effectiveness of known photocatalysts for dye degradation can be maximized by exploring new manufacturing methods.

Due to its properties, cobalt has the potential to provide a three-dimensional transition-metal element with seven three-dimensionally spaced electrons. Its high-spin (HS) state has three unpaired electrons, while its low-spin (LS) state has one. Thus, the electronic structure enhances the activity by structural distortion and lattice orientation control.^17^ Among the oxides, tricobalt tetraoxide (Co_3_O_4_) is a black antiferromagnetic p-type semiconductor, due to its tetrahedral (Td) sites, which are used by Co^2+^ ions, and octahedral (Oh) sites, which are filled by Co^3+^ ions. With superior characteristics, such as electrochemical properties that allow reversible Co^2+^ ↔ Co^3+^ ↔ Co^4+^ redox transitions, it offers a substantial amount of charge storage capacity,^18^ can be used for gas sensing,^19^ has a high catalytic activity,^20^ and has been researched for its applications in many fields, including medical diagnostics.^21^ Most research focuses on the type of nanomaterial used, rather than whether different preparation methods might alter their effectiveness.

Several methods have been studied for the synthesis of nanoparticles, including co-precipitation, sol–gel, and solid-state processes, and hydrothermal procedures.^22^ Hydrothermal synthesis is a solution-based reaction technique used to create nanomaterials that allows control of their morphology.^23^ It is a safe and simple technique for producing nanoparticles at lower temperatures, allowing catalyst growth in a straightforward process.^24^ Inexpensive, uniform reaction conditions provide a small size dispersion.^25^ The structural development of metal oxide nanomaterials is largely controlled by hydrothermal preparation factors, especially reaction times.^26,27^ The electrical structure and photocatalytic performance of the produced materials may be affected by variations in hydrothermal synthesis time, which can control crystal formation, growth, surface morphology, crystallinity, and defect development.^28^ According to recent research, charge-transport behavior and photocatalytic performance may be greatly enhanced by precisely controlling the surface and structural characteristics of metal oxide photocatalysts. It has also been observed that creating functional heterostructures and modifying Co_3_O_4_-based structures afford useful methods for improving catalytic performance and adjusting electrical characteristics.^29,30^ A previous study focused on the impact of synthesis conditions and preparation time on the material. Co_3_O_4_ morphology was found to be highly sensitive to reaction duration, synthesis temperature, and compositional changes. A microflower-like structure was observed in Co_3_O_4_ samples prepared at a comparatively low temperature of 100 °C for 4 h. This suggests that milder conditions and shorter reaction periods promote the nucleation of tiny crystallites that self-assemble into hierarchical, flower-like morphologies.^20^ A broccoli-like morphology was produced after adding multi-walled carbon nanotubes (MWCNTs) and heating for five hours at 150 °C. This suggests that CNTs operate as nucleation templates, limiting the direction of Co_3_O_4_ development and creating more compact, evenly dispersed structures. The product's sheet-like shape that was formed at 140 °C for 6 h was caused by regulated anisotropic crystal formation, which is preferred at intermediate temperatures and moderate reaction times.^31^ Co_3_O_4_ samples exhibited sheet-like structures following 6, 12, and 24 h of NP preparation at 140 °C, which led to the formation of Co_3_O_4_ crystals in two dimensions at moderate temperatures and reaction times, encouraging the formation of thin nanosheets rather than spherical or aggregated particles.^32^

This study aims to assess the photocatalytic degradation performance of Co_3_O_4_ NPs formed using varying hydrothermal durations under low-temperature conditions. Although Co_3_O_4_ is a well-known photocatalytic material for dye degradation, the influence of hydrothermal duration as a key synthesis parameter on the structural evolution, surface properties, and photocatalytic activity of Co_3_O_4_ remains insufficiently explored. As a result, this work shows how regulated preparation conditions might improve the photocatalytic effectiveness of Co_3_O_4_ NPs by systematically examining the link between hydrothermal treatment duration and their physicochemical properties. The modified Co_3_O_4_ nanostructure demonstrated superior stability and recyclability, as well as enhanced degradation performance toward organic dyes, underscoring the significance of synthesis-time optimization for creating effective Co_3_O_4_-based photocatalysts at different hydrothermal durations (12–72 h). Moreover, changes in crystallinity, morphology, and surface characteristics affect charge separation and radical formation during the degradation of EY and BF dyes. Using effective analysis methods, *e.g.* XRD, FTIR spectroscopy, SEM, EDX, and DRS techniques, the structural and morphological properties of the prepared NPs were examined. Finally, the production method, structural alterations, and the ensuing photocatalytic efficiency were directly linked. The study sheds light on the crucial role that preparation design plays in maximizing the performance of oxide-based photocatalysts for environmental applications.

## 2 Materials and methods

### 2.1 Materials

Cobalt nitrate hexahydrate (Co(NH_2_)_2_·6H_2_O, 98%) (PioChem, Egypt), urea (CO(NH_2_)_2_; BioChemica), and polyvinylpyrrolidone were purchased from Alpha Chemika, Germany. Sodium chloride (NaCl), sodium nitrate (NaNO_3_), ascorbic acid (C_6_H_8_O_6_), and isopropyl alcohol (C_3_H_8_O) were purchased from Merck, Germany. Eosin yellow dye was purchased from Henan Alpha Chemika, China. Basic Fuchsin microstain dye was obtained from LOBA, India. Double-distilled water (DDW) was used for further steps.

### 2.2 Preparation of Co_3_O_4_ NPs

**Fig. 1:**
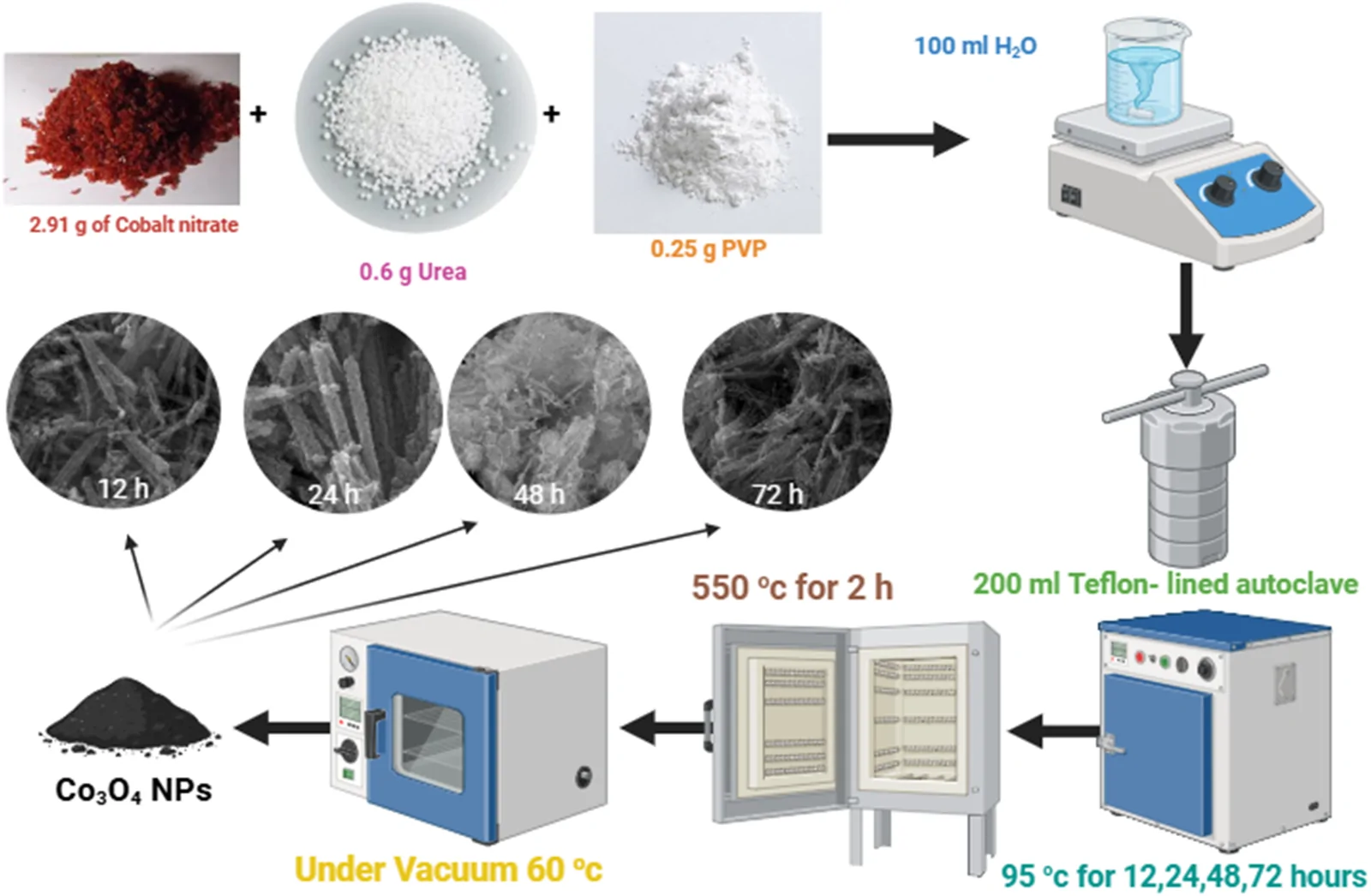
Schematic of the synthesis route of Co_3_O_4_ NPs.

To prepare Co_3_O_4_*via* the hydrothermal approach, 2.91 g of Co(NO_3_)_2_·6H_2_O, 0.6 g of CO (NH_2_)_2_·6H_2_O, and 0.25 g PVP precursors were dissolved in 100 mL of distilled water at room temperature for one hour. The mixture was stirred continuously for a further 30 min, then the mixed dispersion was transferred to a 250 mL autoclave and heated at 95 °C for different times (12, 24, 48, and 72 h), using a hydrothermal parallel synthesizer. The resulting pink powders were collected by centrifugation and then successively washed many times with deionized water and ethanol. The washed powder was then dried under vacuum at 60 °C. The dried powder was then converted into Co_3_O_4_ by heating at 550 °C for 2 h in air with a ramping rate of 10 °C min^−1^. Finally, Co_3_O_4_ was produced by calcining the obtained black powders in a muffle furnace at 550 °C for 2 h (Fig. 1).

### 2.3 Photocatalytic measurements

The photocatalytic activity of the Co_3_O_4_ NPs was examined using 50 mL of dye solution. The photocatalyst was added to the beaker in a 0.01 g dose. To achieve adsorption–desorption equilibrium, EY and BF dye concentrations (each 0.005 g L^−1^ with initial pH of 7 and 6.8, respectively) were combined for 30 min in the dark. The reactor was built at ECCM Com, Egypt, and has several safety measures. A 75-mW cm^−2^ solar light was used for the experiments, and the solution beaker was placed 12 cm above the ground. A UV-vis spectrophotometer (X-ma 1000, Human Corporation) was used to analyze the samples over a wavelength range of 400–800 nm. Over the course of the exposure, samples (3 mL) were taken every 30 min for 260 min. To remove the catalyst, the samples were centrifuged for 10 min at 5000 rpm. UV-vis spectroscopy was then used to filter and examine the solutions, with the degradation efficiency calculated as follows:1
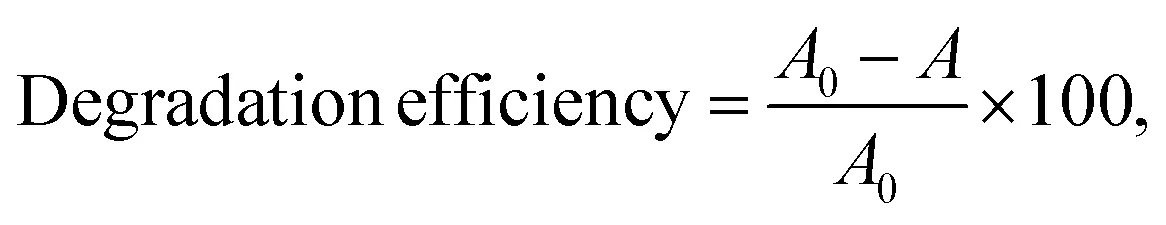
where *A* is the optical absorption at the required time, and *A*_0_ is the initial dye absorbance. The maximum of the absorption spectra for EY and BF dyes were measured at 517 and 546 nm, respectively, to determine all absorbance values. The solution detailed above was then exposed to simulated solar light under identical photocatalytic reaction working conditions. The fit regression model tool in the Minitab software was used for regression analysis of the kinetics models. Based on statistical metrics such as the regression coefficient (*R*^2^), modified *R*^2^, and error analysis, a series of kinetic models were assessed to identify the best-fitting model.

### 2.4 Instrumental characterization

Functional groups were examined using an FT-IR spectrophotometer (Nicolet6700, Thermo Electron Corporation, Waltham, MA) within *ν* 4000–400 cm^−1^. XRD (Bruker, Germany) with a step size of 0.02° and a 2*θ* range of 10°–80° using Cu Kα1 was used for structural analysis. Using X'pert High Score program, X-ray diffraction peaks of the samples were indexed. UV-vis spectra were recorded using a JASCO V-650 double-beam spectrophotometer equipped with a single monochromatic beam and a photomultiplier tube detector, with BaSO_4_ serving as the reference. SEM-EDX was utilized to investigate the morphology and elemental content of the fabricated materials. SEM samples were first coated with a gold (Au) coating at a voltage of 30 kV before investigation; elemental mapping was performed using a Vega 3 SBU instrument (Tescan, Czech Republic). The Brunauer–Emmett–Teller (BET) surface areas of the samples were obtained by monitoring the N_2_ adsorption and desorption of Co_3_O_4_ NPs at 77 K (BELSORP-miniX 10 115). Optical scattered reflectance measurements at *λ* 200–700 nm were conducted using a Shimadzu UV-3600 (Japan) UV-vis spectrophotometer.

## 3 Results and discussion

### 3.1 X-ray analysis

**Fig. 2:**
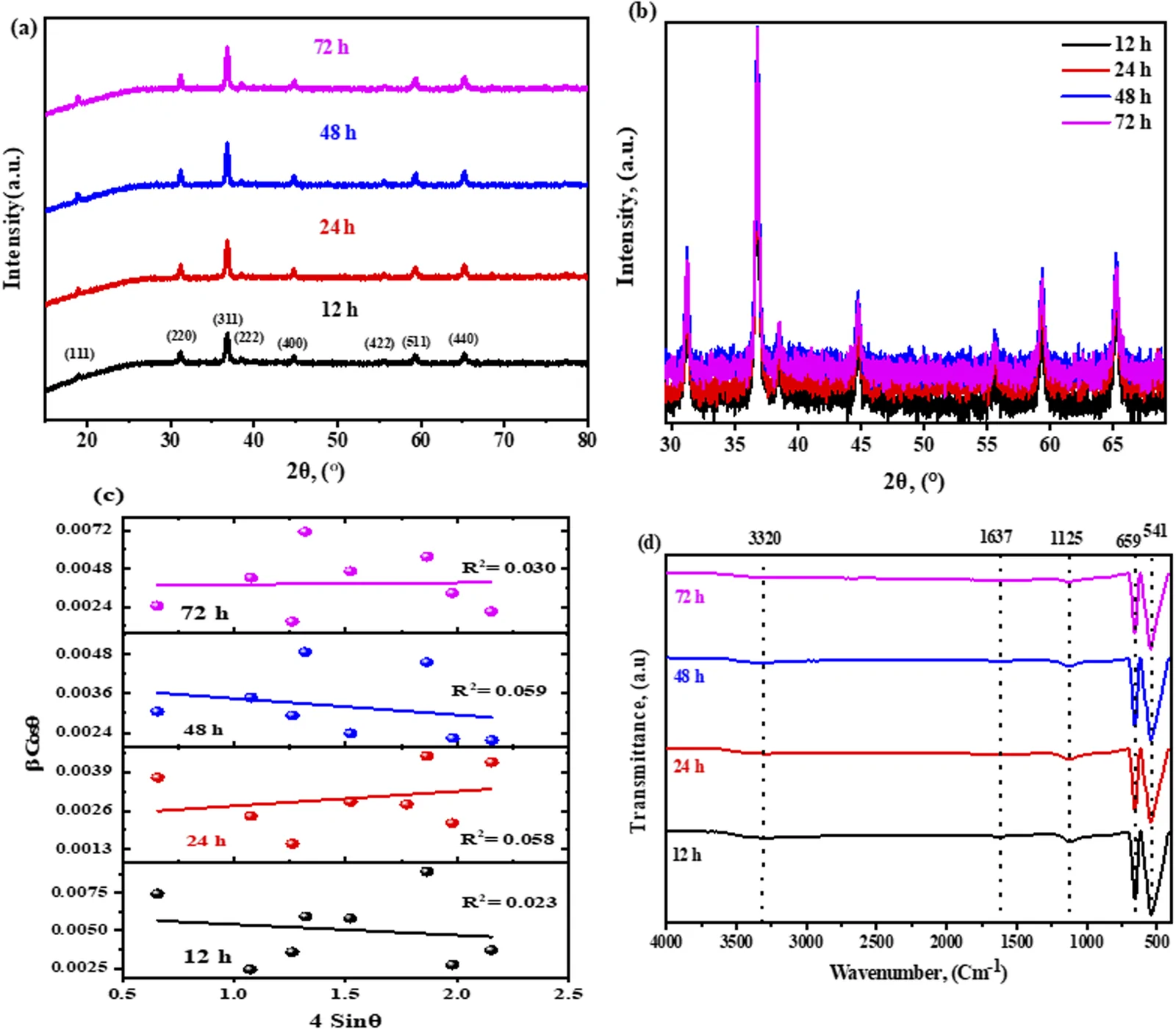
(a) XRD patterns of Co_3_O_4_ NPs prepared with different hydrothermal reaction times, (b) stacked XRD patterns, and (c) Williamson–Hall analysis. (d) FTIR spectra of Co_3_O_4_ NPs prepared with different hydrothermal reaction times.

Fig. 2a shows the XRD patterns of Co_3_O_4_ formed with different synthesis times, using 12 h Co_3_O_4_ as a reference. XRD patterns of Co_3_O_4_ exhibit characteristic peaks at 2*θ* = 18.92°, 31.11°, 36.75°, 38.59°, 44.69°, 55.57°, 59.28°, and 65.14°, corresponding to the (*hkl*) planes of (111), (220), (311), (222), (400), (422), (511), and (440), respectively,^33,34^ for cubic Co_3_O_4_ (card No. 00-009-0418). From the XRD results, it was found that the synthesized tricobalt tetraoxide diffraction pattern did not contain additional peaks; *i.e.*, the different synthesis-time Co_3_O_4_ samples did not exhibit additional diffraction peaks compared with the 12 h Co_3_O_4_ sample, as seen in Fig. 2b. In contrast, their crystallite sizes increased with increasing reaction time. The average crystallite sizes of the fabricated NPs were determined using the Scherrer formula as follows:2
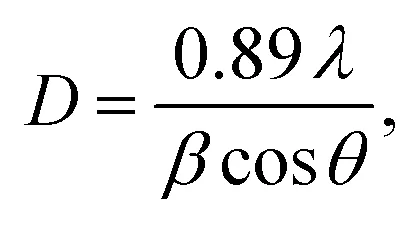
where *D* is the average crystallite size, *λ* is the X-ray wavelength (0.154 nm), *θ* is the Bragg angle, and *β* is the FWHM. The average crystallite size of the 12 h Co_3_O_4_ NPs was found to be 33.22 nm. However, the size is influenced by the fabrication method. The calcination of materials at a specific temperature alters the dipole moments of the polar molecules at a constant temperature.^35^ This process affects molecular distribution and friction, thereby resulting in NPs with variable particle sizes.^36^ The uniform deformation model is used in the Williamson–Hall analysis^37^ as follows:3
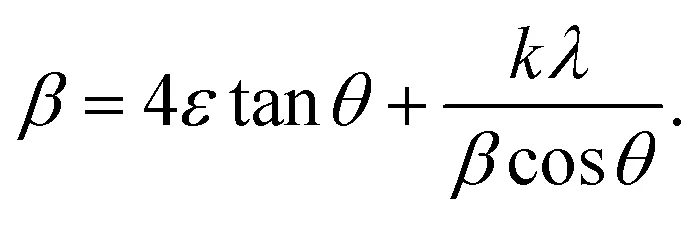
In Fig. 2c, lattice strain (*ε*) and crystallite size (*D*) of each sample are calculated by computing the slope of the linear plot of *β* cos *θ vs.* 4 sin *θ*, with the *y*-intercept relating to *kλ D*. Table 1 displays crystallite size and lattice strain values that were determined in this way. As reaction time increases, the hydrothermal dislocation and lattice strain increase compared to those of the 12 h Co_3_O_4_. Williamson–Hall (W–H) analysis and the Scherrer equation were both used to determine the microcrystal size of the Co_3_O_4_ samples. Microcrystal size grew between 12 and 24 h in both procedures, reaching a maximum value of *D* = 51.52 nm and *C*_s_ = 60.40 nm as a result of enhanced crystal development. The microcrystal size decreased slightly with additional autoclave time to *D* = 46.9758 nm and *C*_s_ = 34.711392 nm at 48 h Co_3_O_4_, suggesting restricted crystal development and the potential for recrystallization.^38^ Texture coefficients (TC_*hkl*_) were utilised to indicate the degree of orientation, a parameter that characterises the growing anisotropy as follows:4
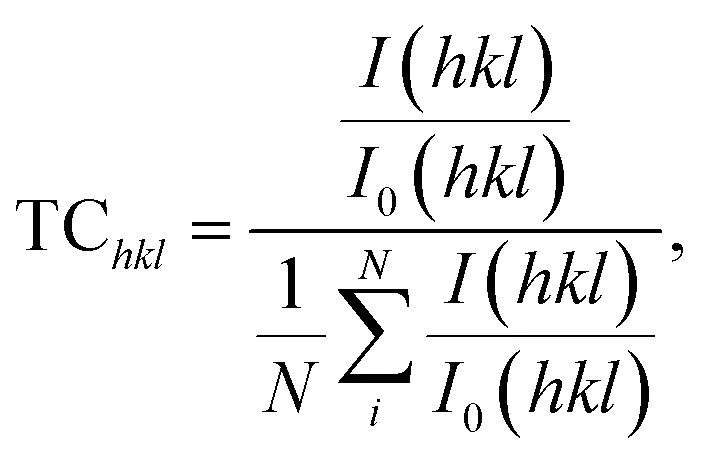
where TC_*hkl*_ is the texture coefficient of plane (*hkl*), *I* is the measured intensity of each (*hkl*) *n* peak, *I*_0_ is the theoretical relative intensity according to the JCPDS-ICDD card, and *N* is the number of reflections.^39^ Since we chose the three strongest reflections, *i.e.*, the (220), (311), and (440) peaks, *N* = 3 in this instance.

**Table 1:** Lattice parameters, *e.g.* average crystallite sizes (*C*_s_ & *D*), mean lattice strain, and mean dislocation density, of the Co_3_O_4_ photocatalysts

Sample	*C*_s_ W–H (nm)	*D* (nm)	Mean dislocation (*δ*)	Mean lattice strain (*ε*)
Co_3_O_4_ (12 h)	22.403594	33.22437	1.580 × 10^−3^	1.265 × 10^−3^
Co_3_O_4_ (24 h)	60.400881	51.51686	5.247 × 10^−4^	7.565 × 10^−4^
Co_3_O_4_ (48 h)	34.711392	46.9758	5.833 × 10^−4^	8.018 × 10^−4^
Co_3_O_4_ (72 h)	37.157181	45.48279	9.400 × 10^−4^	9.660 × 10^−4^

Table 2 displays the computed TC values for the three main diffraction planes of Co_3_O_4_. The T_*hkl*_ values for the 48 h Co_3_O_4_ nanocomposite are obviously higher than those of the 12 h Co_3_O_4_. The three planes in the nanocomposite of 48 h Co_3_O_4_ appear to be more oriented than those of the 12 h Co_3_O_4_, based on this finding. It is noted that the intensity of the (220) diffraction peak is noticeably higher than that of the other diffraction peaks. Then it decreased slightly as the hydrothermal reaction time increased to 72 h. This suggests that the polar faces of Co_3_O_4_ might be altered during the hydrothermal synthesis as the hydrothermal reaction time increased from 4 to 7 h.^40^ This finding indicates that 48 h Co_3_O_4_ NPs have a substantial (220) preferred growth orientation, indicating that the hydrothermal duration also had a considerable impact on the crystallographic orientation of Co_3_O_4_ NPs.

**Table 2:** Texture coefficient parameters of the Co_3_O_4_ NPs prepared with different hydrothermal reaction times

NPs formed with different hydrothermal reaction times	Diffraction planes	Intensity (a.u)		Calculated TC_hkl_ for 3 main peaks	FWHM (radian)	Peak position (degrees)
		*I*_0_ (JCPDS-ICDD00-009-0418)	*I* (measured)			
Co_3_O_4_ (12 h)	(220)	40	31.51	0.93	0.002512	31.11
	(311)	100	100	1.18	0.003768	36.75
	(440)	45	33.62	0.88	0.004396	65.14
Co_3_O_4_ (24 h)	(220)	40	35.96	1	0.002512	31.20
	(311)	100	100	1.11	0.00157	36.79
	(440)	45	35.68	0.88	0.005024	65.19
Co_3_O_4_ (48 h)	(220)	40	34.90	1.01	0.003604022	31.23
	(311)	100	100	1.16	0.003089411	36.81
	(440)	45	31.37	0.81	0.0025748	65.23
Co_3_O_4_ (72 h)	(220)	40	27.77	0.88	0.004396	31.23
	(311)	100	100	1.44	0.00157	36.77
	(440)	45	29.78	0.95	0.002512	65.12

### 3.2 FTIR analysis

The FTIR spectra of Co_3_O_4_ samples, shown in Fig. 2d, reveal five bands located at *ν* 3320, 1637, 1125, 659, and 541 cm^−1^. Labelling of these Raman bands was made based on available published reports.^41^ Two strong absorption peaks were detected in the FT-IR spectra of virgin Co_3_O_4_ at *ν* 541 and 659 cm^−1^, which are ascribed to the O–Co–O bond's bridging vibration and Co–O stretching vibration.^42^ Moreover, the peak at *ν* 1125 cm^−1^ links to C–O stretching, revealing residual organic fragments, likely from incomplete breakdown of acetate.^43^ Surface hydroxyl groups (–OH) were indicated by the absorption bands in the *ν* 1637 cm^−1^ region,^44^ whereas peaks at *ν* 3220 cm^−1^ were assumed to be due to adsorbed water molecules.^45^Fig. 2d shows an obvious decrease in peak intensity as the reaction time increases, indicating that the water content gradually decreases with longer reaction times. All Co^3+^ ions in the prepared Co_3_O_4_ sample occupied octahedral sites, while Co^2+^ ions were in tetrahedral sites, indicating that the sample adopted a typical spinel structure.^46^

### 3.3 SEM analysis

**Fig. 3:**
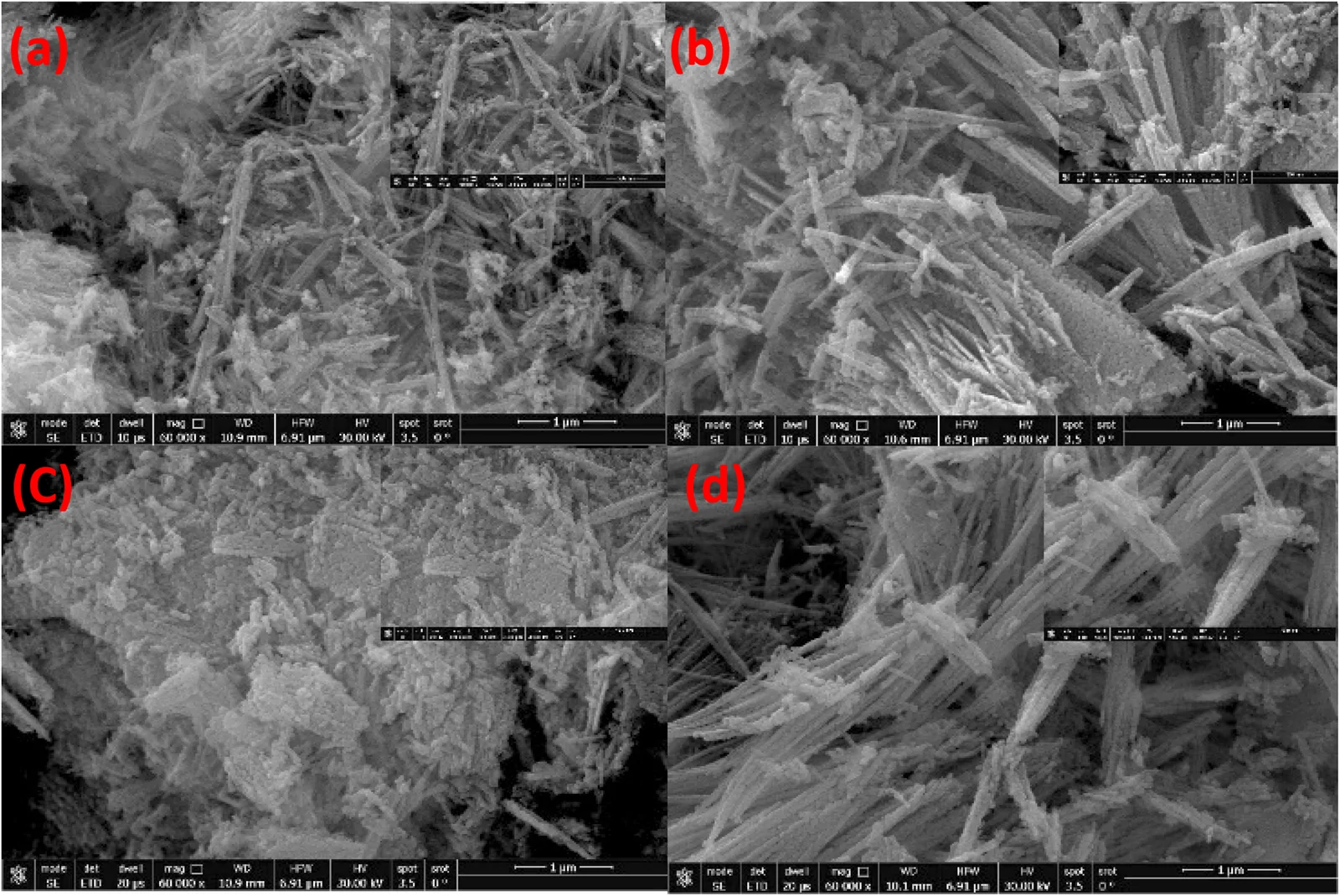
(a–d) SEM images of Co_3_O_4_ NPs prepared with varied autoclave (hydrothermal) times from 12–72 h.

**Fig. 4:**
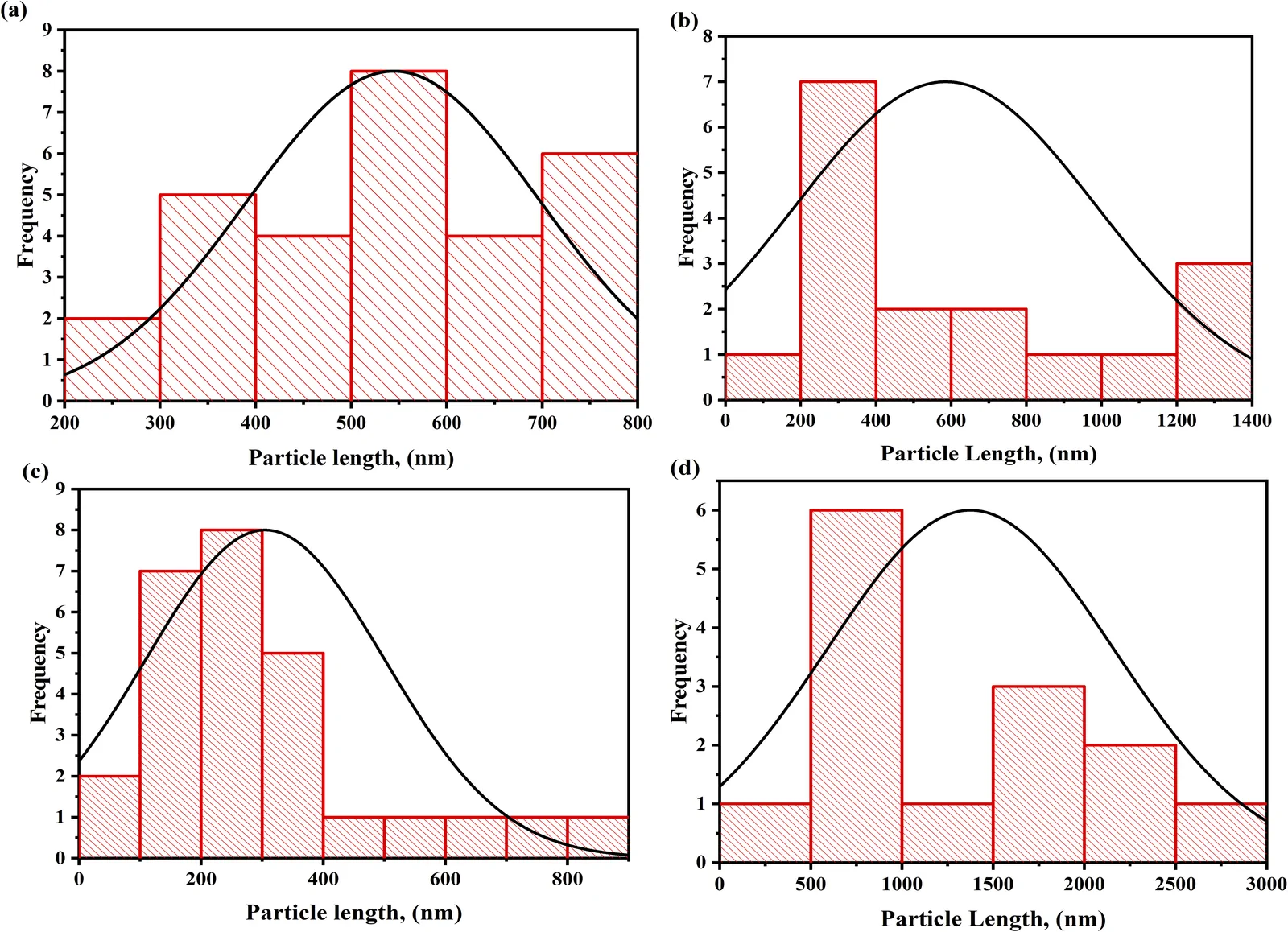
Particle length distribution histogram models of (a) 12 h Co_3_O_4_, (b) 24 h Co_3_O_4_, (c) 48 h Co_3_O_4_, and (d) 72 h Co_3_O_4_ NPs.

**Fig. 5:**
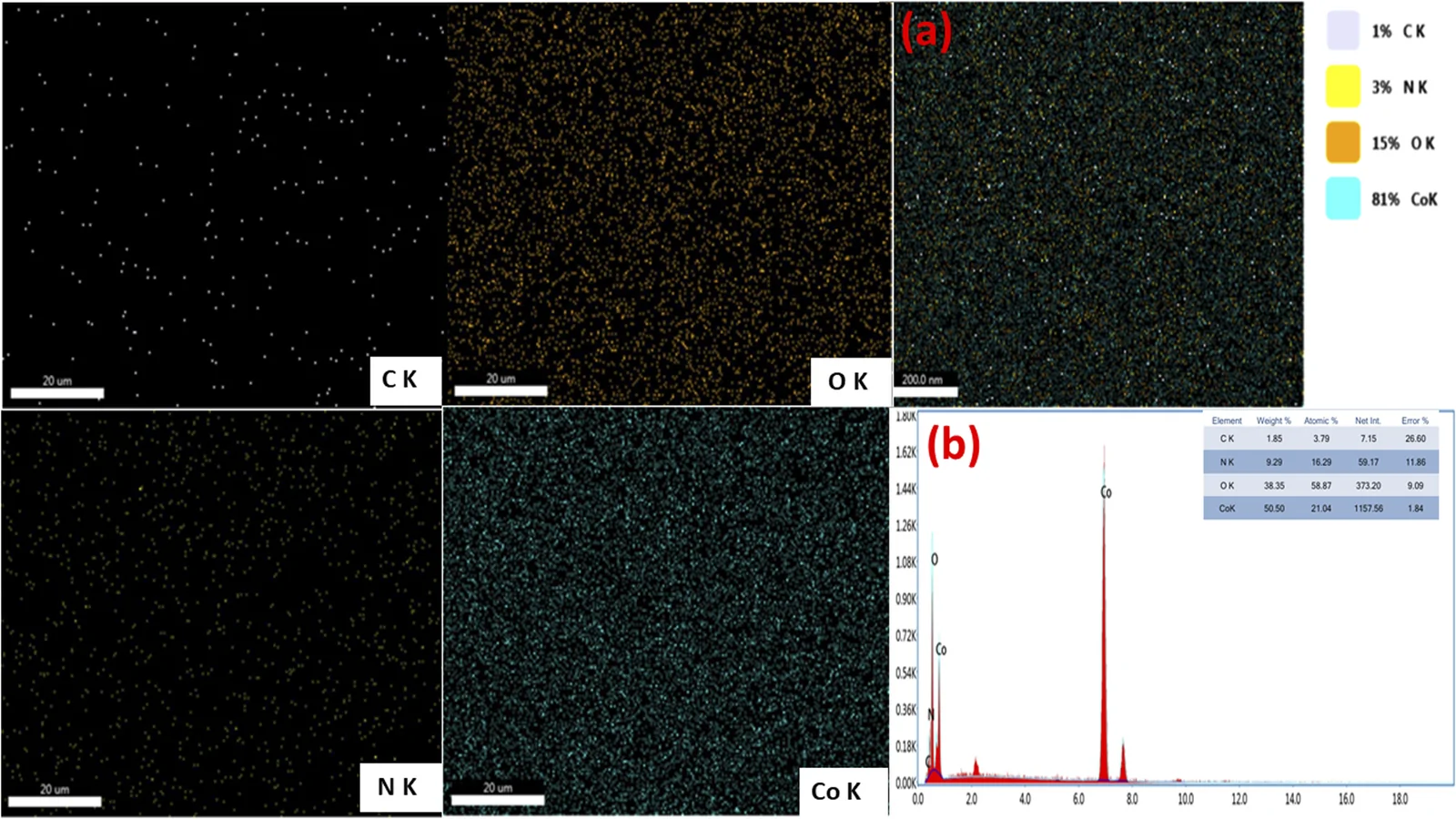
(a) Elemental mapping images of Co_3_O_4_ NPs and (b) EDX spectrum of Co_3_O_4_ NPs.

The preparation method plays a crucial role in determining the structural, morphological, physical, and chemical properties of the Co_3_O_4_ particles. Variations in preparation conditions, particularly the calcination time, significantly influence particle growth, granularity, agglomeration, and surface roughness, as observed through SEM analysis. These structural modifications significantly affect the optical and catalytic behavior of the catalyst.^47^Fig. 3(a–d) show SEM images of 12 h Co_3_O_4_, 24 h Co_3_O_4_, 48 h Co_3_O_4_, and 72 h Co_3_O_4_ NPs. As can be observed, the resulting Co_3_O_4_ has a needle-like appearance. The morphology evolved from short needle-like aggregates (12 h and 24 h) to more compact and densely packed needle-like structures (48 h), followed by the re-emergence of elongated needle-like features at 72 h. In particular, the structures of the short needle-like Co_3_O_4_ and their morphology after 24 h and 72 h are essentially the same, as shown in Fig. 3(c and d). One possible explanation for the morphological shift is the required time for preparation. Table 3, comparing the effects of different synthesis methods on the morphology of the prepared samples, clearly shows that changing the preparation process results in variations in the final structure, with preparation technique resulting in different morphologies, suggesting that the preparation conditions have a significant impact on the material's structural morphology. The total number of particles with lengths of different nanoparticle sizes in the range of 18–188 nm is shown as histogram plots in Fig. 4a–d. The largest aspect ratios are found in the particle size range of 24 nm (Table 4). These elements are uniformly distributed, suggesting Co_3_O_4_ homogeneity (Fig. 5a). The EDX spectrum shows a distinct signal for Co at 7 keV and O at 0.5 keV, confirming the preparation of very pure Co_3_O_4_ NPs.^48^ Co_3_O_4_ elemental mapping images (Fig. 5b) show that the sample comprises Co and O. In contrast, SEM-EDX elemental mapping images reveal the newly added elements C and N, which are attributed to PVP and urea introduced during the production process.^49,50^

**Table 3:** Effects of different synthesis methods on produced Co_3_O_4_ NPs properties

Synthesis method	Morphology	Particle size (nm)	Band gap (eV)	Ref.
Sol–gel	Aggregation	NA	2.77	51
Hydrothermal	Multi-layered	2000–3000	1.89	52
Hydrothermal	Ultrathin sheets	NA	NA	53
Hydrothermal	Crystal sheets	120	NA	54
Hydrothermal	Nanoflowers	26	NA	55
Co-precipitation	Agglomerated and granular	10–20	NA	56
Sucrose template	Cubic and hexagonal	33.68	NA	57
Scallion root template	Tubular	11.6	NA	58
MOF-template	Nanocubes	5–8	NA	59
Hydrothermal	Nanoneedles	32–48	3.46	Present work

**Table 4:** Average particle lengths, widths, and aspect ratios (L/W) of the Co_3_O_4_ NPs prepared with different hydrothermal times, as determined by SEM analysis

Sample	Mean length (nm)	Mean width (nm)	Mean aspect ratio
12 h	±18.12927	±2.493567	±12.43285
24 h	±77.90918	±8.465353	±7.163151
48 h	±33.16015	±2.377885	±5.478881
72 h	±188.9818	±5.617692	±24.03221

### 3.4 DRS analysis

**Fig. 6:**
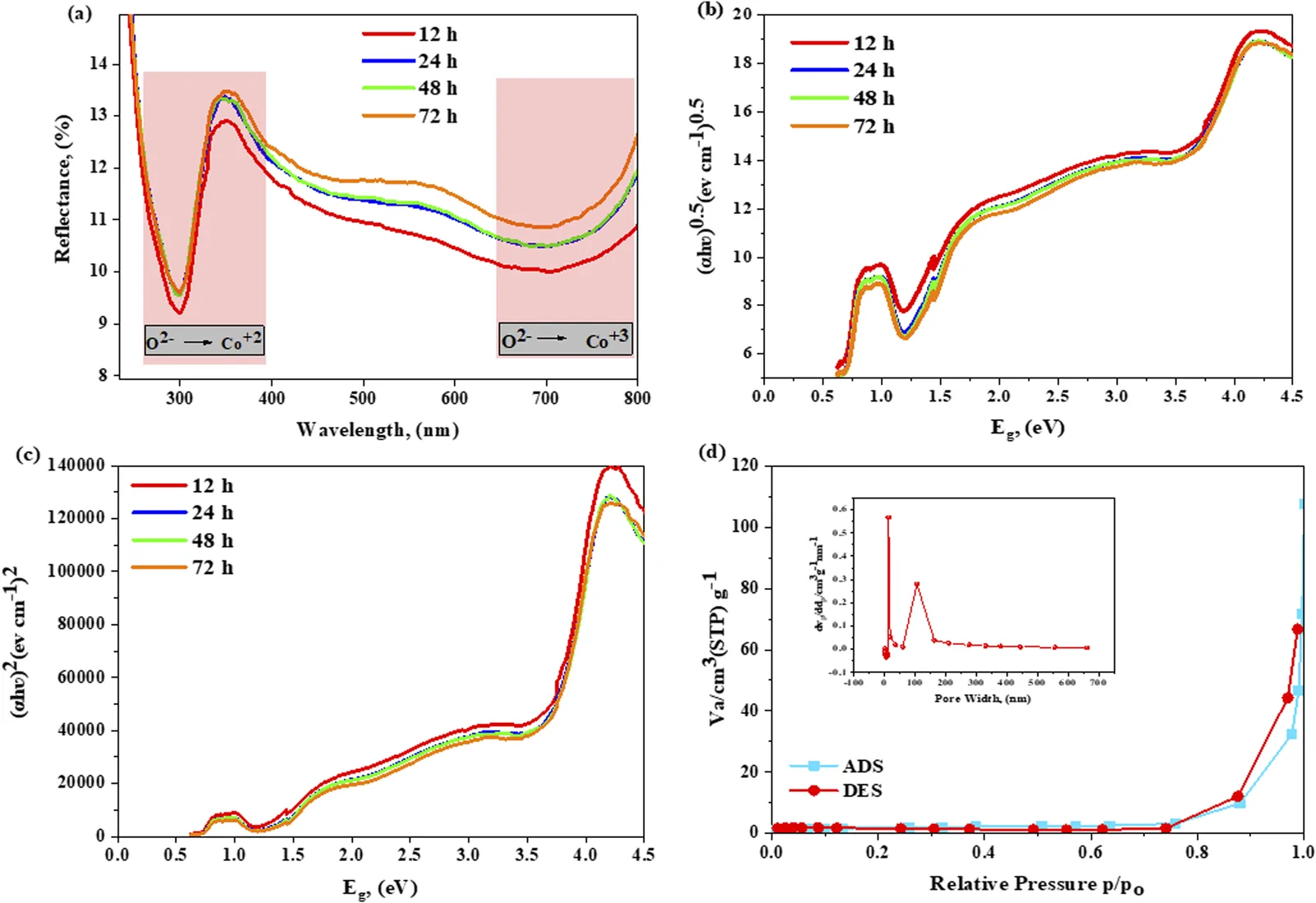
(a) UV-vis DRS spectra. Tauc plots for the (b) indirect and (c) direct band gap transitions of the synthesized Co_3_O_4_ NPs. (d) N_2_ adsorption–desorption isotherms and BJH pore distributions of the 48 h Co_3_O_4_ sample.

DRS analyses of samples of 12 h Co_3_O_4_, 24 h Co_3_O_4_, 48 h Co_3_O_4_, and 72 h Co_3_O_4_ are shown in Fig. 6, which displays a detailed exploration of the analytical findings and their connection to photocatalytic performance. The primary objectives are to enhance our understanding of the mechanisms underlying photocatalytic reactions in these systems and to identify the key elements that influence catalytic efficiency. Thus, UV-vis absorption spectroscopy was used to determine the energy gap (*E*_g_) required for electron excitation from the valence band to the conduction band during the photocatalysis process.

Fig. 6a displays the UV-vis spectra of Co_3_O_4_ and its NPs, which are recorded at *λ* 295–283 nm. Moreover, the charge transfer from O to Co orbitals was clarified by two broad peaks. The movement of Co^2+^ and Co^3+^ orbital electrons towards O^2−^ shows the double oxidation state of cobalt elements.^60^ Tauc's equation was used to determine the energy band gaps as follows:

5 (*αhv*) = *A*(*hv* − *E*_g_)^*n*^

The optical band gap (*E*_*g*_) can be found by projecting the Tauc plot, which is the linear part of the (*αhv*)^2^ against *hν* plot, where *A* is a proportionality constant and *n* is an exponent that varies according to the type of electronic transition (*n* = 0.5 for direct transitions and *n* = 2 for direct transitions), *α* is the absorption coefficient, and *hν* is the photon energy. Tauc's equation states that *E*_g_ is the tangent line's intercept with the energy axis. Photocatalytic processes can be improved, and more potent catalysts formed, by closely examining the DRS spectra and investigating the structural, morphological, and electrical characteristics of nanomaterials.^61^Fig. 6(b and c) show the direct and indirect band gap energies of Co_3_O_4_ NPs. This is attributed to O^2−^ → Co^2+^ and O^2−^ → Co^3+^ charge-transfer processes. Bhargava *et al.*^62^ stated that Co_3_O_4_ has different band gaps, which were found between 1.66 and 2.12 eV. The existence of two band gaps implies that the fabricated samples are pure, p-type semiconductors. Likewise, the band gap value is found to decline with increasing reaction temperature as the crystallite size increases.^63^ However, all of the Co_3_O_4_ samples exhibit both direct and indirect transitions.^64^ While the *E*_g_ indirect may be related to defect/indirect transition levels, we primarily concentrate on the higher *E*_g_ direct in our study, as it governs the material's photocatalytic/optical performance. In contrast, 12 h Co_3_O_4_ has a bandgap of 3.53 eV, resulting in lower photocatalytic activity compared to that of 48 h Co_3_O_4_ (3.46 eV) (Table 5) when exposed to solar light. In addition to crystallite growth from 33.22 to 46.98 nm and a drop in lattice strain from 1.265 × 10^−3^ to 8.018 × 10^−1^, the direct band gap decreased from 3.53 eV (12 h) to 3.46 eV (48 h), suggesting structural rearrangement during hydrothermal treatment. The electronic band structure may change as a result of such structural changes, which affect defect-related localized states.^65^ It is commonly known that defect states alter the optical transitions and add energy levels inside the band gap.^66^ Despite the almost constant crystallite size (45.48 nm), the band gap marginally increased to 3.51 eV after additional treatment for 72 h, indicating that the observed fluctuation is related to ongoing defect formation and lattice rearrangement rather than grain size alone, by the shift in lattice strain from 8.018 × 10^−1^ to 9.660 × 10^−2^.^67^

**Table 5:** Calculated optical band gap values of the prepared Co_3_O_4_ NPs showing both their direct and indirect transitions

Sample	*E*_g_ indirect (eV)	*E*_g_ direct (eV)
Co_3_O_4_ (12 h)	2.46	3.53
Co_3_O_4_ (24 h)	2.37	3.48
Co_3_O_4_ (48 h)	2.33	3.46
Co_3_O_4_ (72 h)	2.45	3.51

### 3.5 Surface area and porosity studies

From BET analysis, N_2_-adsorption/desorption isotherms, and BJH analysis were used to assess the material's surface characteristics. In particular, the specific surface area and pore characteristics of the Co_3_O_4_ sample synthesised with 48 h autoclave time were assessed (Fig. 6d). A macroporous structure was indicated by a type IV isotherm with an H3-type hysteresis loop, which yielded a specific surface area of 6.5035 m^2^ g^−1^, a total pore volume of 0.1615 cm^3^ g^−1^, and an average pore diameter of 112 nm. The decrease in surface area may be explained by the longer autoclave/hydrothermal treatment period, which probably encouraged particle development and aggregation and decreased the accessible surface area. The surface-to-volume ratio reduces as a result of longer reaction durations, which also promotes crystal formation and lowers the percentage of nanoparticles.^68^ This sample had the largest surface area, compared to samples made with shorter or longer autoclave times, demonstrating the critical role that autoclave time plays in regulating porosity and particle development. The well-controlled nucleation and crystal formation under ideal hydrothermal conditions, resulting in smaller particle sizes and more accessible surface sites, are responsible for the increased surface area. These findings demonstrate that modifying the autoclave duration successfully enhances the textural characteristics of Co_3_O_4_, making this preparation technique superior to traditional methods as documented elsewhere.^69,70^

### 3.6 Photocatalytic activity

**Fig. 7:**
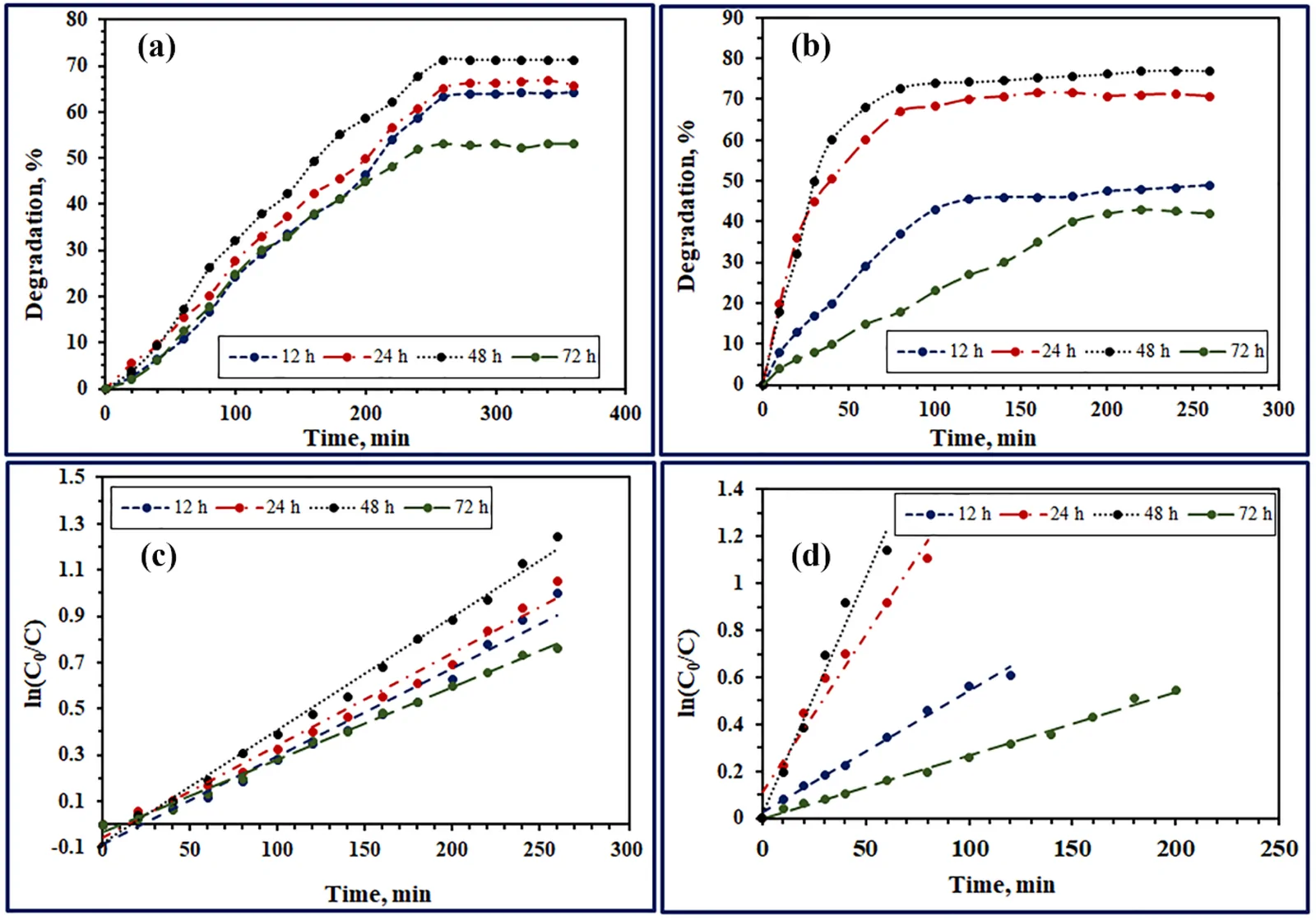
Degradation efficiencies of the (a) eosin yellow dye and (b) basic Fuchsin dye. Kinetic studies of the (c) eosin yellow dye and (d) basic Fuchsin dye.

Fig. 7 shows the photocatalytic degradation of 50 mL solutions of 5 ppm of eosin yellow (EY) and basic Fuchsin (BF) by Co_3_O_4_ NPs under simulated solar light. Continuous degradation of EY and BF is indicated by the decreasing intensity of the absorption peaks over time, as shown in Fig. S1 and S2 (see the SI). Distinct absorption bands are observed at 517 and 546 nm. All samples were processed under the same conditions, with a 20 min interval between recordings to assess the photodegradation process of EY and BF.

In the dark, catalysis tests were conducted using EY and BF to determine the adsorption–desorption equilibrium, which required this pre-irradiation phase. A more noticeable drop in absorbance after 30 min in the dark indicates that 48 h Co_3_O_4_, as seen in Fig. 7, exhibits more adsorption activity than 12 h Co_3_O_4_. However, when exposed to simulated solar light, the 48 h Co_3_O_4_ photocatalyst exhibits a sharper and faster decrease in absorbance, indicating higher photocatalytic effectiveness. For the 48 h Co_3_O_4_ sample, increased light absorption, decreased recombination losses, and improved charge-carrier separation are likely responsible for this improved performance. Under solar illumination, eosin yellow gradually degrades with 260 min of exposure to 48 h Co_3_O_4_, with 71.2% of EY being photodegraded (Fig. 7a). The degradation of BF reaches 76.9%, as shown in Fig. 7b. However, Co_3_O_4_ significantly boosts photocatalytic activity at various synthesis times, highlighting that the synthesis duration is key to improving eosin yellow degradation. The 48 h Co_3_O_4_ catalyst shows the highest activity for the degradation of both dyes, with improved charge separation and extended spectral response resulting from the 48 h hydrothermal treatment. The following equation was used to determine the pseudo-first-order kinetics, which are shown by linear correlation in the insets of Fig. 7(c and d):6
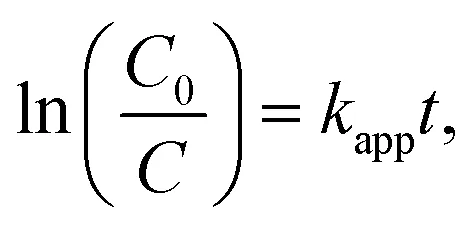
7
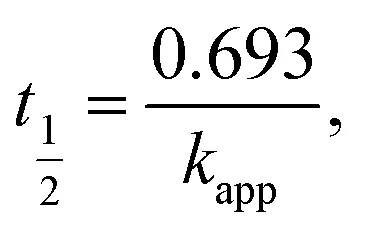
where *k* represents the kinetic rate, *t* is the time, *C* and *C*_0_ are the concentrations at time *t* and the initial concentration, respectively, for the EY or BF dyes, and *t* = *t*_1/2_ is the half-life time of the reactions calculated from *k*; the results are shown in Table 6. It is confirmed that 48 h Co_3_O_4_ has the highest reaction rate (*k* = 0.0049 min^−1^ for EY and *k* = 0.02 min^−1^ for BF), suggesting that it might be a useful photocatalyst for photodegradation of both anionic and cationic dyes and pollutants under simulated solar light.

**Table 6:** Photocatalytic degradation efficiencies of dyes and their kinetic parameters

Catalyst at different times	Deg (%)		*k*_app_ (min^−1^)			*R* ^2^			*t*_0.5_ (min)	
	EY	BF	EY	BF	BF (stat)	EY	BF	BF	EY	BF
12 h	64.26386	48.9298	±0.0038	±0.0052	0.00515	0.98188	0.99488	0.991	182.3684	133.2692
24 h	65.78307	70.70523	±0.004	±0.0134	0.01339	0.99206	0.97954	0.963	173.25	51.71642
48 h	71.26362	76.93378	±0.0049	±0.02	0.01998	0.9934	0.99136	0.976	141.4286	34.65
72 h	53.20308028	41.99961	±0.0031	±0.0027	0.00268	0.99691	0.9983	0.995	223.5484	256.6667

### 3.7 Pseudo first-order kinetic statistical assessment of basic Fuchsin dye

**Fig. 8:**
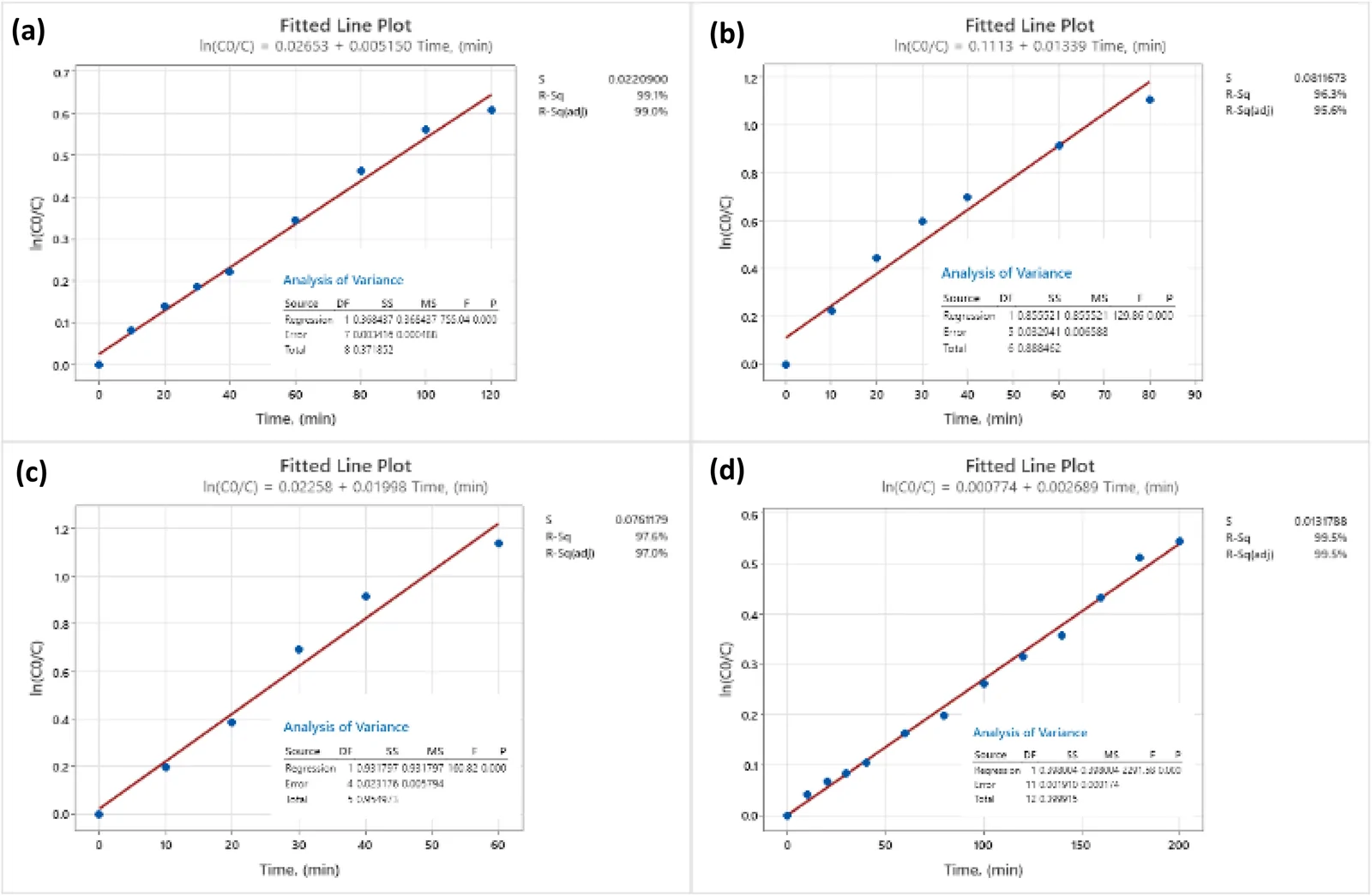
Linear fitting of ln (*C*_0_/*C*_*t*_) *versus* the irradiation time for the (a) 12 h, (b) 24 h, (c) 48 h, and (d) 72 h Co_3_O_4_ NPs using a pseudo-first-order kinetic model.

Regression method and statistical fitting techniques differed across Minitab investigations, resulting in a divergence in the kinetic constants obtained. In contrast to traditional graphical kinetic fitting, Minitab provided statistically verified fitting. The results of the kinetics study on Co_3_O_4_ samples for BF dye degradation, reaction rate, and correlation coefficients *R*^2^ are shown in Fig. 8(a–d). These results were compared with those of the conventional process to determine the difference in outcomes. The actual results were matched to a quadratic polynomial using the Minitab software, which was used to predict the optimum point for four catalyst autoclave times. The irradiation time in min is represented by the independent variable (*t*). The apparent rate constants (*k* = 0.005150 min^−1^, *k* = 0.01339 min^−1^, *k* = 0.01998 min^−1^, and *k* = 0.002689 min^−1^), as shown in Table 6, which indicate the response rate of dye degradation, are represented by the slope of the linear fit. The initial adsorption effects and departure from ideal kinetic behaviour at *t* = 0 are reflected in the intercept. The least-squares fitting process is the source of the modest non-zero intercept found in the linear regression, which represents slight experimental errors rather than any extra adsorption change. According to ANOVA, the regression model is statistically significant for 12 h, 24 h, 48 h, and 72 h (*F* = 755.04, *F* = 129.86, *F* = 160.82, and *F* = 2291.58, respectively, with *P* = 0.00).^71^ The observed connection is not the result of random variation, as shown by the low *p*-value. On the other hand, residual error indicates small variances that can be attributed to surface-related or experimental variables. However, Table 7 compares the estimated Co_3_O_4_ photocatalyst parameters with those reported in the literature for Co_3_O_4_ with different preparation techniques. The 48 h Co_3_O_4_ photocatalyst shows higher degradation at a lower dose without additives compared with that at a higher dose and no additives.

**Table 7:** Estimated photocatalyst parameters such as pollutant type, additive, dose, pollutant concentration, lamp source and degradation values of pure Co_3_O_4_ compared with the reported data for different synthesis techniques

Pollutant	Additive	Dose (g L^−1^)	*C*_0_ (g L^−1^)	Time	Lamp source	Degradation (%)	Ref.
Phenol	PMS	0.2	0.02	90	Halide lamp	100	77
TC	—	0.5	0.01	150	Xe lamp	20	78
MB	H_2_O_2_	0.5	0.01	60	UV-C lamp	79	79
MB	—	1	0.0025	120	UV lamp	10	80
MB	H_2_O_2_	0.6	0.01	60	Xe lamp	91.72	81
MB	H_2_O_2_	0.6	0.025	200	Hg lamp	100	82
CR	H_2_O_2_	0.6	0.05	60	UV lamp	90	83
EY and BF	—	0.01	0.005	260	Solar lamp	71% and 78%	Present work

### 3.8 Photocatalytic degradation mechanism and trapping test

**Fig. 9:**
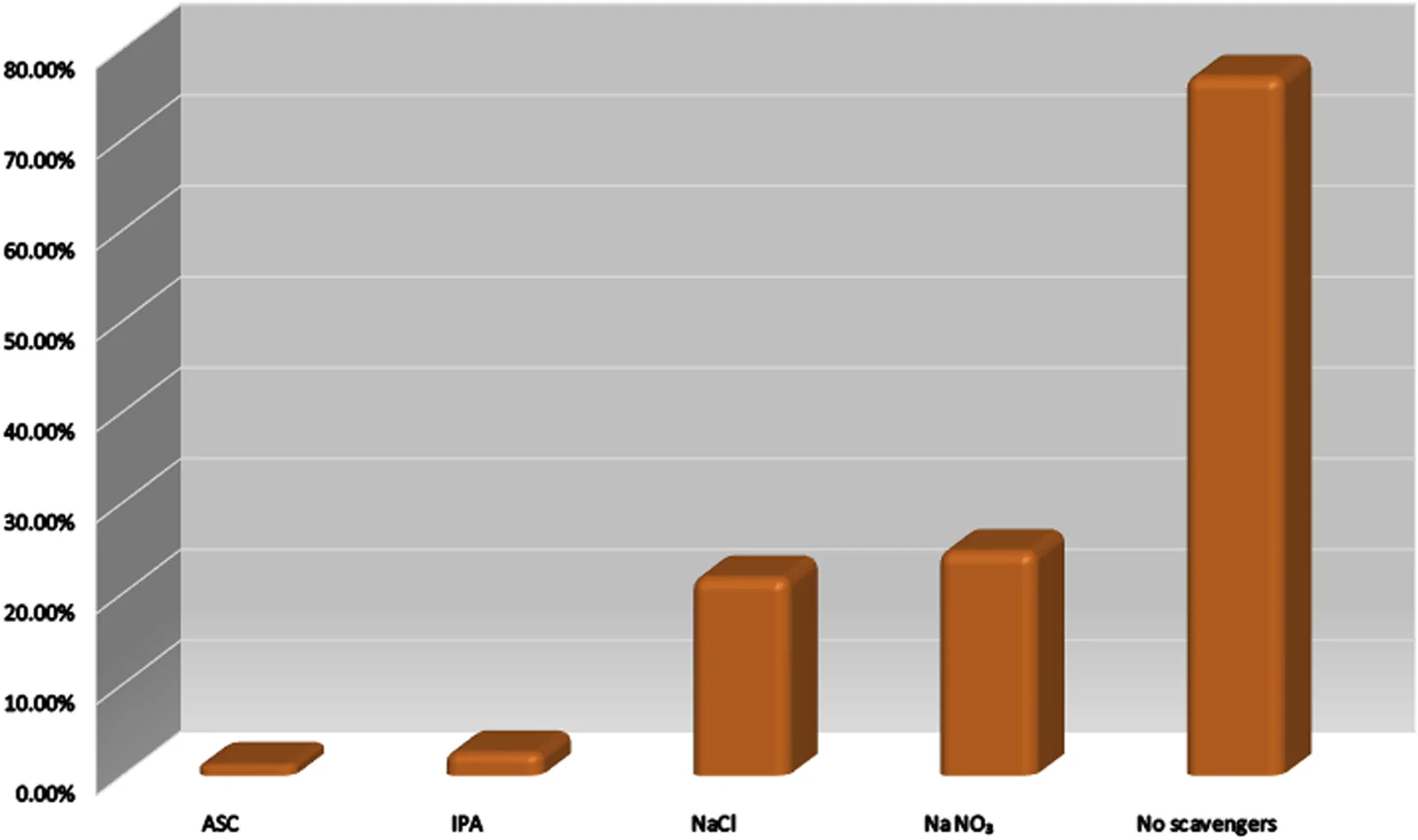
Scavenger tests for BF dye degradation using the 48 h Co_3_O_4_ photocatalyst.

Several radical scavengers, including ascorbic acid [superoxide radicals (O_2_˙^−^)], isopropyl alcohol [hydroxyl radicals (˙OH)], sodium chloride [holes (h^+^)], and sodium nitrate [electrons (e^−^)], were used to scavenge radical species during the photocatalytic process of BF degradation (Fig. 9). The scavenger trapping experiments were conducted under the same conditions used for the photocatalytic experiments. Under ideal conditions, around 78% of the basic Fuchsin dye was degraded in 260 min without using scavengers. When a photocatalyst is present, the inclusion of ASC or IPA scavengers, which trap the superoxide anion radical O˙^−^ and ˙OH radicals, respectively, reduced the degradation to around 2.5% and 1.2%, respectively. The inclusion of sodium nitrate as an e-quencher reduced dye degradation to about 24.7%. Following the use of sodium chloride, an h^+^ scavenger, there was a 21.8% reduction in the elimination of basic Fuchsin dye.^72^ The radical trapping tests confirmed that the main reactive species causing dye degradation are hydroxyl radicals (˙OH) and superoxide radicals (O_2_˙^−^).^73^

**Fig. 10:**
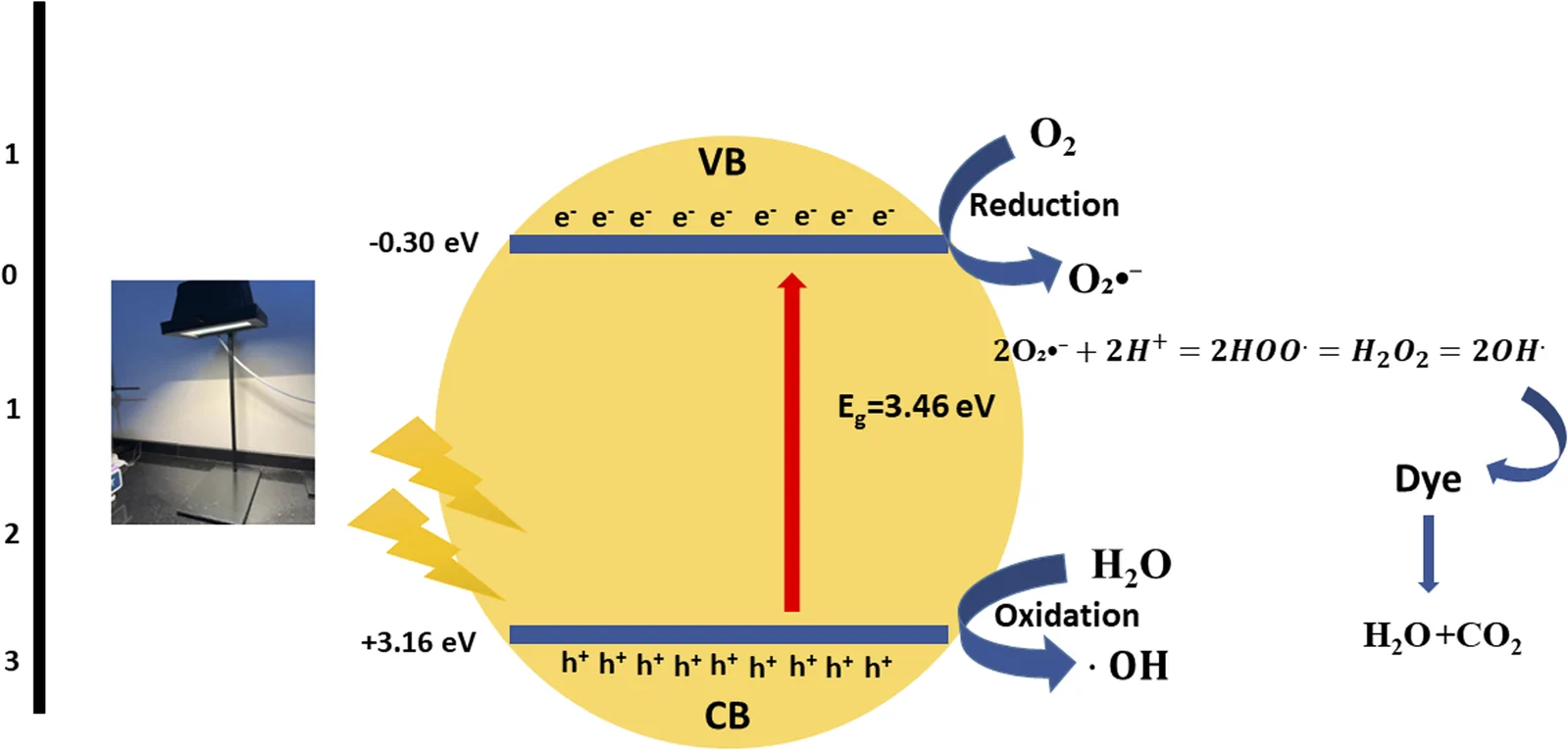
Schematic of the photoactivity mechanism upon solar-light illumination of the Co_3_O_4_ NPs.

As depicted in Fig. 10, under simulated solar light irradiation of 48 h Co_3_O_4_ NPs, absorbable photons with energy equal to or higher than the band gap (3.46 eV) excite electrons in the valence band to the conduction band to generate electron–hole pairs^74^ as follows:

8 Co_3_O_4_ + *hv* → h^+^ + e^−^.

Photogenerated holes accumulated in the valence band react with surface hydroxyl groups or water molecules to generate a hydroxyl radical as follows:

9 OH^−^ + h^+^ → ˙OH,

10 H_2_O + h^+^(6) + → ˙OH + h^+^.

In the meantime, superoxide anion radicals (O_2_˙^−^) are created when excited electrons (e^−^) react with dissolved oxygen molecules. These radicals can then combine with protons to produce hydroperoxyl radicals 
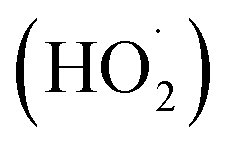
. Together, these reactive oxygen species (ROS), which include ˙OH, O_2_˙^−^, and 
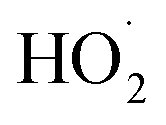
, enhance the photocatalytic breakdown of pollutants (Fig. 10)^75^ as follows:11
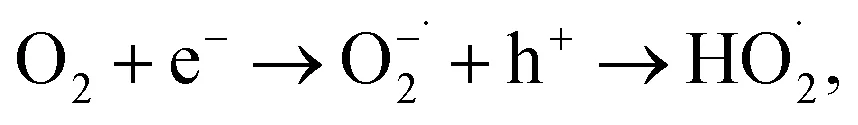


The following equations were used to calculate the *C*_B_ and *V*_B_ bands as follows:*E*_CB_ = *X* − *E*_C_ − 0.5 *E*_g_,*E*_VB_ = *E*_CB_ + *E*_g_,where *X* (5.93 eV), *E*_C_, and *E*_g_ represent the absolute electronegativity, band gap energy, and free electron energy (4.5 eV), respectively, of 48 h Co_3_O_4_.^76^ These characteristics were used to calculate the valence band (*V*_B_ = +3.16 eV) and conduction band (*C*_B_ = −0.30 eV) of 48 h Co_3_O_4_.

### 3.9 Photocatalytic recyclability

**Fig. 11:**
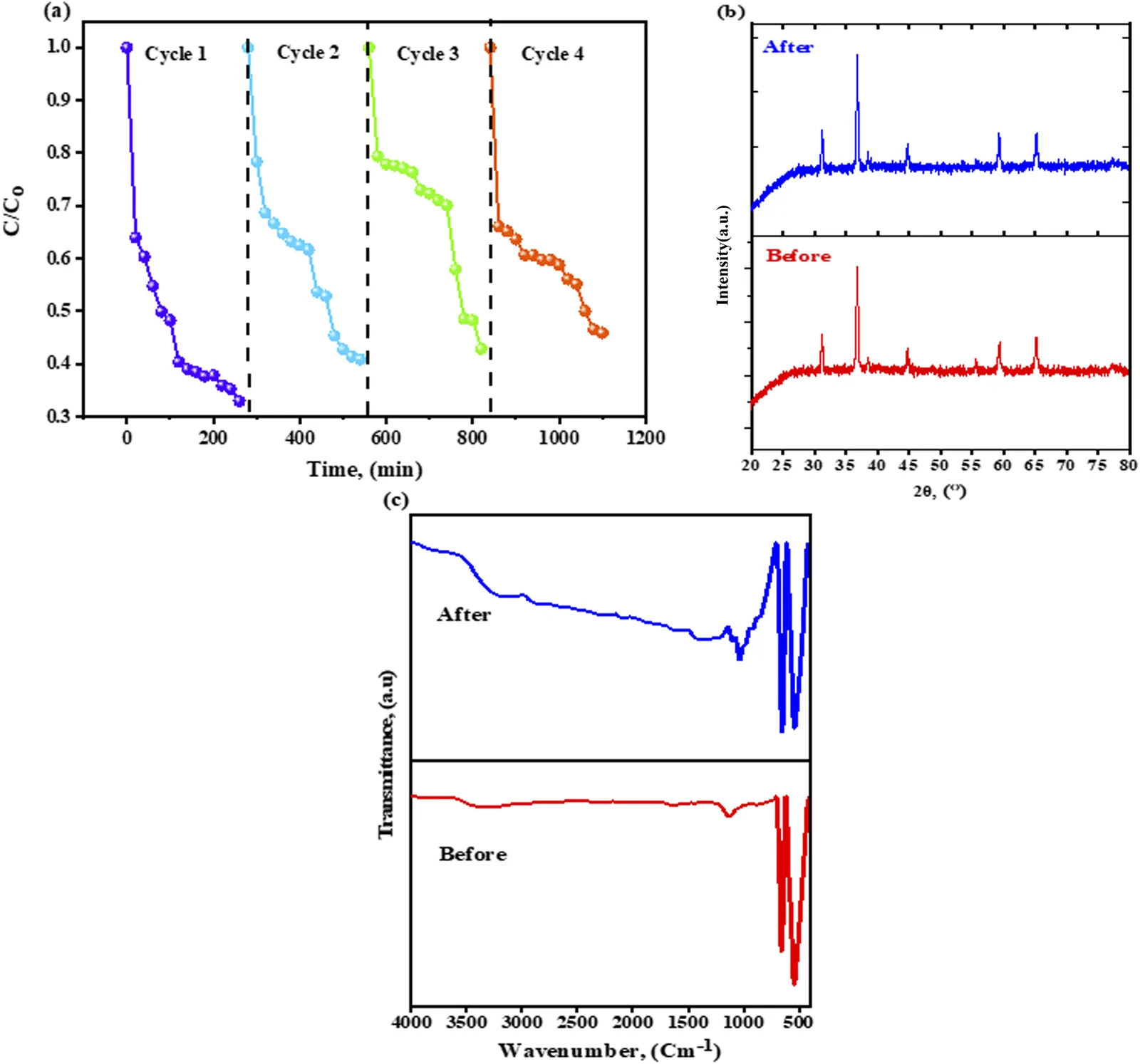
(a) Reusability study of the 48 h Co_3_O_4_ NPs over 4 cycles. (b) XRD analysis after recycling of the 48 h Co_3_O_4_ NPs. (c) FTIR analysis after recycling of the 48 h Co_3_O_4_ NPs.

Following the degradation process, catalyst particles were filtered out, dried, and then reused to break down additional contaminants. The catalyst's active participation throughout the process was confirmed by repeating this recycling phase for four successive photodegradation cycles. With every run, there was a modest decrease in efficiency (Fig. 11a). Nevertheless, the 48 h Co_3_O_4_ catalyst continued to exhibit a noteworthy degradation efficiency of 54.07% for BF dye even after the fourth cycle, with no sharp initial decrease. These results demonstrate the catalyst's high mechanical durability and reliability after repeated usage. Furthermore, XRD and FTIR results for the recycled 48 h Co_3_O_4_ sample show no changes compared with the original sample (Fig. 11b and c), demonstrating its exceptional resistance to BF destruction. The nanosized 48 h Co_3_O_4_ is therefore proposed as an efficient and competitive photocatalytic compound to treat wastewater using solar energy due to its exceptional photocatalytic capacity and reusability.

## 4 Conclusions

This study demonstrated that the hydrothermal reaction time plays a crucial role in regulating the physicochemical properties of needle-like Co_3_O_4_ nanoparticles prepared at a constant temperature. Increasing the reaction time from 12 to 48 h improved the crystallinity, phase purity, particle homogeneity, and crystal size, thereby enhancing the photocatalytic performance. The improved Co_3_O_4_ sample synthesized after 48 h exhibited a direct energy band gap of 3.46 eV, a BET surface area of 6.5 m^2^ g^−1^, and the highest photolysis efficiencies of 71% for eosin yellow (EY) and 76% for basic Fuchsin (BF) under simulated solar irradiation. Trapping experiments confirmed that superoxide radicals (O_2_˙^−^) and hydroxyl radicals (˙OH) are predominant reactive species responsible for the dye degradation. Furthermore, the improved photocatalyst exhibited good reusability, with only a slight decrease in photocatalytic activity after four consecutive cycles, demonstrating its satisfactory stability for heterogeneous photocatalytic applications. These results confirm that controlling the hydrothermal reaction time is an effective strategy for enhancing the structural, optical, and photocatalytic properties of pure cobalt oxide nanoparticles for wastewater treatment applications.

## Author contributions

Habiba A. Hossni: methodology, investigation, formal analysis, data curation. Amany S. El-Khouly, M. Y. Nassar, and Rasha Abu-Khudir: methodology, investigation, funding acquisition, formal analysis. Heba Y. Zahran: writing – original draft, visualization, validation. Elbadawy A. Kamoun: writing – review and editing, writing – original draft, visualization, validation, supervision, project administration. V. Ganesh: software, resources, methodology, investigation. Ibrahim S. Yahia: writing – review and editing, writing – original draft, supervision, software, resources, project administration.

## Conflicts of interest

The authors declare that they have no known competing financial interests or personal relationships that could have appeared to influence the work reported in this paper.

## Funding

This work was supported by the Deanship of Scientific Research, the Vice Presidency for Graduate Studies and Scientific Research, King Faisal University, Saudi Arabia [Project No. KFU254578].

## Supplementary Material

NA-OLF-D6NA00411C-s001

## Data Availability

The data sets used and/or analyzed during the current study are available from the corresponding authors upon reasonable request. Supplementary information (SI) is available. See DOI: https://doi.org/10.1039/d6na00411c.
