# Supplementary material for: Tuning crystallinity and electronic structure of Co3O4 nanoparticles via hydrothermal time for improved photocatalytic dye degradation and statistical kinetic evaluation
Source: Nanoscale Adv. 2026 Sep 7;8(20):6056–72. doi: 10.1039/d6na00411c (PMC13595296; doi:10.1039/d6na00411c)
Supplement: Supplementary file 1 [file NA-008-D6NA00411C-s001.pdf]

Supplementary Information (SI) for Nanoscale Advances.
This journal is © The Royal Society of Chemistry 2026

Supplementary materials

Tuning Crystallinity and Electronic Structure of Co_3O_4 Nanoparticles *via* Hydrothermal Time for Improved Photocatalytic Dye Degradation and Statistical Kinetic Evaluation

Elbadawy A. Kamoun[a*], Habiba A. Hossni[b], V. Ganesh[c], Mohammed A. Alkhalifah[a], M.Y. Nassar[a], Esam M. Bakir[a], Heba Y. Zahran[c], Ibrahim S. Yahia[c*]

[a] Department of Chemistry, College of Science, King Faisal University, Al-Ahsa 31982, Saudi Arabia.
[b]Nanotechnology Section, Egyptian Company for Carbon Materials, El-Sheraton/El-Nozha, Cairo, 11757, Egypt.
[c]Laboratory of Nano-Smart Materials for Science and Technology (LNSMST), Department of Physics, Faculty of Science, King Khalid University, P.O. Box 9004, Abha, Saudi Arabia.

**Coresponding authors: ekamoun@kfu.edu.sa and badawykamoun@yahoo.com (E.A. Kamoun), and dr_isyahia@yahoo.com (I.S. Yahia).*

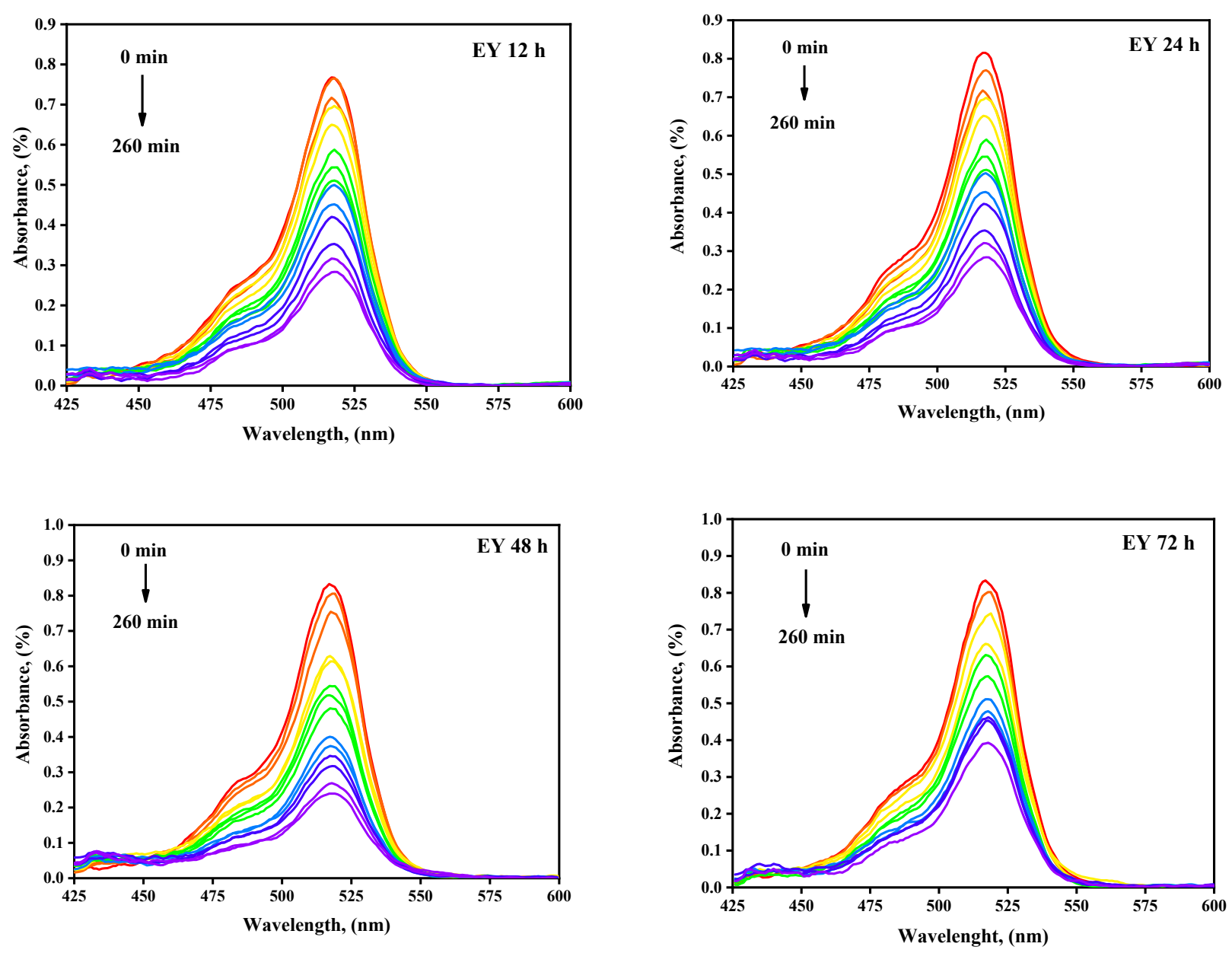

Fig. S1 UV-Vis. absorption spectra of Co_3O_4 in solar light degradation of eosin yellow EY dye over different times of calcination.

Supplementary materials

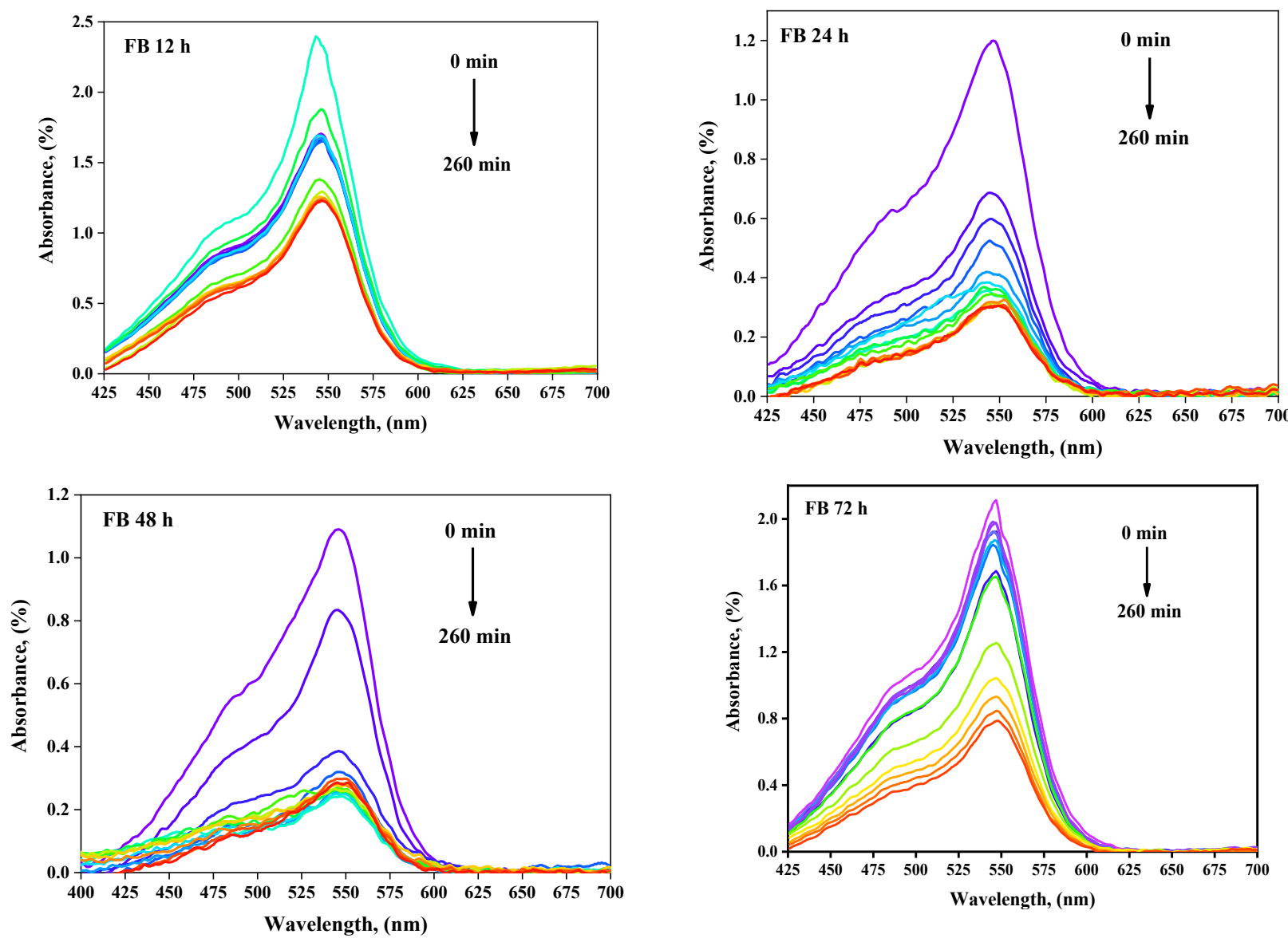

Fig. S2 UV-Vis. absorption spectra of Co_3O_4 in solar light degradation of Fusion Basic FB dye over different times of calcination.
